# Glucose-6-phosphate dehydrogenase at the crossroads of redox homeostasis and regulated cell death in erythrocytes

**DOI:** 10.3389/fphar.2026.1958205

**Published:** 2026-10-26

**Authors:** Nuriye Nuray Ulusu

**Affiliations:** 1 Department of Medical Biochemistry, School of Medicine, Koç University, Istanbul, Türkiye; 2 Koç University Research Center for Translational Medicine (KUTTAM), Istanbul, Türkiye

**Keywords:** eryptosis, ferroptosis, glucose-6-phosphate dehydrogenase, hemolytic anemia, NADPH metabolism, pentose phosphate pathway, precision medicine, programmed cell death

## Abstract

Glucose-6-phosphate dehydrogenase (G6PD) provides the sole source of NADPH in mature erythrocytes, fueling the cell’s essential antioxidant defenses. Although G6PD deficiency is traditionally tied to acute oxidative hemolysis, red cell loss extends beyond simple membrane rupture. This perspective evaluates G6PD-dependent NADPH supply as a metabolic redox checkpoint that controls the balance between erythrocyte survival, regulated cell death, and clearance. Reduced NADPH availability can limit glutathione regeneration and thus decrease GPX4-dependent lipid peroxide detoxification. Although this biochemical relationship provides a possible link between redox imbalance and erythrocyte injury, the direct involvement of ferroptosis and necroptosis-like pathways in mature erythrocytes remains insufficiently proven. Acute hemolysis, in this view, is a downstream consequence of these regulated events rather than the primary cause. G6PD is essential for erythrocyte survival as their sole source of NADPH. G6PD deficiency alone does not always cause cell death, but under oxidative stress, it acts as a metabolic checkpoint that shifts red blood cells from survival to regulated death rather than simple osmotic lysis.

## Introduction

Glucose-6-phosphate dehydrogenase (G6PD, glucose-6-phosphate (G6P): NADP^+^ oxidoreductase, EC 1.1.1.49) is the rate-limiting enzyme of the pentose phosphate pathway (PPP) (Tandogan et al., 2011; Ulusu, 2024). G6PD deficiency is the most common X-linked enzymatic disorder affecting cellular defense against oxidative stress. Beyond ROS scavenging, the concomitant depletion of NADPH disrupts essential reductive biosynthetic processes and impairs reduced glutathione regeneration required for xenobiotic detoxification (Aydemir and Ulusu, 2023). G6PD deficiency has long been known as a hemolytic disorder associated with recurrent hemolytic anemia (Beutler, 1994). Altered G6PD activity bridges cellular metabolic imbalances with diverse pathological conditions, highlighting its dual role in promoting oncogenic progression upon hyperactivity and accelerating degenerative or endocrine disorders upon impairment (Karaman and Ulusu, 2024). The depletion of intracellular NADPH can lead to severe pathological consequences by impairing both antioxidant defenses and NADPH-dependent metabolic processes (Ulusu, 2026).

In addition to inherited G6PD deficiency, acquired impairment of G6PD activity has been reported in metabolic and hematological disorders, including diabetes mellitus and chronic myelomonocytic leukemia. Although the underlying causes differ, both inherited and acquired G6PD deficiency share reduced NADPH generation, impaired antioxidant defense, and increased sensitivity to regulated cell death (Gök et al., 2016; Naville et al., 2022; Pes and Dore, 2022). G6PD deficiency is traditionally categorized as a conditional hemolytic syndrome. Yet, it fundamentally represents a chronic, systemic disruption of cellular redox homeostasis that could serve as a key bridge to diverse programmed cell death pathways and broader organ pathologies (Garcia et al., 2021).

The classical view of G6PD-associated erythrocyte loss largely focuses on passive membrane rupture. However, erythrocytes can undergo regulated cell death or programmed clearance before complete hemolysis occurs, suggesting that red cell loss involves multiple, mechanistically distinct processes.

This perspective examines the potential links between G6PD deficiency, redox imbalance, and distinct pathways of erythrocyte loss. It distinguishes well-established evidence for eryptosis and macrophage-mediated clearance from more tentative evidence linking NADPH depletion to ferroptosis-like lipid injury and erythronecroptosis. While this perspective article does not contain primary experimental data, it evaluates current literature to support G6PD as a key metabolic redox checkpoint in mature erythrocytes. Specifically, this work outlines how G6PD-dependent NADPH supply sustains GSH levels (Turan et al., 2001; Aydemir and Ulusu, 2023), maintains GPX4-dependent lipid peroxide clearance (Xie et al., 2023; Zheng et al., 2024) and prevents premature divergence toward distinct regulated cell death pathways, including eryptosis (Lang and Lang, 2015; Bouguerra G, 2021), ferroptosis-like lipid injury (Cao et al., 2021; Stephenson et al., 2025), and erythronecroptosis (Tkachenko and Havranek, 2025).

G6PD is essential for erythrocyte survival as their sole source of NADPH. When G6PD is deficient, red blood cells do not simply undergo osmotic lysis; under oxidative stress, this deficiency acts as a metabolic checkpoint that guides a controlled switch from survival to regulated cell death.

## Diseases that cause acquired G6PD enzyme deficiency

Unlike congenital X-linked G6PD deficiency, high-glucose-induced G6PD impairment is an acquired post-translational downregulation (Pes and Dore, 2022). Hyperglycemia activates the cAMP/PKA pathway, leading to the inhibitory phosphorylation of G6PD, which dramatically reduces its enzymatic activity despite normal protein synthesis (Zhang et al., 2000).

In diabetes, prolonged hyperglycemia reduces G6PD activity through post-translational modification rather than changes in gene expression. High glucose activates protein kinase A (PKA), which phosphorylates G6PD and alters its structure, leading to reduced enzyme activity (Xu et al., 2005). As a result, NADPH production declines, antioxidant defenses become impaired, oxidative stress increases, and cells become more vulnerable to regulated cell death. This mechanism suggests that both inherited and acquired G6PD deficiency ultimately converge on a common pathway of redox imbalance (Zhang et al., 2000; Stanton, 2012).

Severe hemolysis due to acquired G6PD deficiency was observed during the evolution of a hematological malignancy into chronic myelomonocytic leukemia. Genetic analysis confirmed that this acquired defect resulted from the clonal expansion of precursor cells carrying a G6PD null variant (Naville et al., 2022).

## The core paradigm: NADPH scarcity and cellular vulnerability

G6PD converts glucose-6-phosphate to 6-phosphoglucono- δ -lactone, a process coupled with the reduction of NADP^+^ to NADPH. In mature erythrocytes, which lack mitochondria and nuclei, the PPP represents the *sole* source of NADPH. This coenzyme is essential for reducing glutathione ( GSH ) and thioredoxin, the cell’s primary antioxidants against oxidative stress (Wei et al., 2022). Thus, NADPH availability represents a fundamental metabolic determinant of whether erythrocytes maintain redox homeostasis or undergo regulated cell death.

## Deconstructing the death pathways: beyond passive lysis

Cell death is broadly divided into two categories. Accidental cell death (ACD) is an uncontrollable process caused by sudden physical or environmental damage that disrupts membrane integrity. It does not rely on intracellular signaling pathways and cannot be targeted or prevented pharmacologically. But regulated cell death (RCD) develops gradually; the cell has time to trigger protective responses, making these pathways promising targets for drug discovery (Tkachenko et al., 2025).

Red blood cell loss in G6PD deficiency was thought to result from simple osmotic fragility or mechanical breaking inside the spleen. Oxidatively damaged erythrocytes are removed by splenic macrophages in the spleen (Luzzatto et al., 2016). This phagocytic removal is triggered because oxidative stress causes hemoglobin to precipitate into Heinz bodies, which severely reduces cell deformability (Luzzatto et al., 2016). NADPH depletion may represent a molecular switch that links metabolic dysfunction to regulated cell death pathways (Thiagarajan et al., 2021).

## The ferroptotic axis

Ferroptosis is an important form of cell necrosis, and several papers highlighted the critical role of G6PD in preventing ferroptosis (Cao et al., 2021; Chen et al., 2026). Ferroptosis is a form of regulated cell death driven by iron-dependent lipid peroxidation (Čepelak et al., 2020). The depletion of NADPH indirectly compromises the activity of selenoprotein glutathione peroxidase 4 (GPX4), which relies strictly on reduced GSH as its obligate electron donor. NADPH acts upstream to fuel glutathione reductase (GR), the enzyme responsible for regenerating GSH from oxidized glutathione (GSSG). When G6PD activity or NADPH supply is limited, the resulting exhaustion of the GSH pool secondarily impairs GPX4-dependent lipid peroxide clearance rather than directly inactivating the enzyme itself (Tandogan et al., 2011; Xie et al., 2023; Zheng et al., 2024). Without these reducing equivalents, the lipid bilayer undergoes severe peroxidation, which is rapidly driven by the high levels of intracellular free iron. The accumulation of free iron catalyzes harmful reactions and induces ferroptotic cell death (Abdukarimov et al., 2025).

Notably, even in mitochondria-lacking mature erythrocytes, G6PD deficiency can precipitate ferroptosis because upstream NADPH restriction limits GSH regeneration, thereby secondarily disabling GPX4 protection and allowing free iron released from labile hemoglobin pools to directly catalyze lethal plasma membrane lipid peroxidation.

## Eryptosis and macrophage clearance

Eryptosis is the predominant form of regulated cell death in mature erythrocytes and is conventionally characterized by oxidative stress, cytosolic calcium Ca^2+^ influx, and membrane remodeling (Scovino et al., 2022).

In canonical eryptosis, oxidative stress-induced Ca^2+^ influx activates Ca^2+^-dependent calpain proteases, which degrade cytoskeletal proteins such as Band 3 and spectrin, driving membrane micro-blebbing (Lang and Lang, 2015). In G6PD deficiency, the involvement of Ca^2+^-signaling remains complex; while phosphatidylserine externalization is consistently observed, intracellular Ca^2+^ levels are not always elevated at baseline (Gupte et al., 2020). Thus, G6PD-deficient eryptosis may proceed through Ca^2+^-independent oxidative damage pathways (Bouguerra G, 2021). While canonical eryptosis relies heavily on ${\text{Ca}}^{2+}$ entry, eryptosis in G6PD deficiency can also occur via ${\text{Ca}}^{2+}$ -independent pathways, driven primarily by oxidative reactions dependent, cross-linking of membrane proteins and severe energy depletion. Whether operating through ${\text{Ca}}^{2+}$ -dependent or ${\text{Ca}}^{2+}$ -independent mechanisms, these cellular stresses induce erythrocyte shrinkage and trigger phosphatidylserine exposure. Macrophages subsequently recognize this surface-exposed phosphatidylserine to phagocytose and eliminate the compromised erythrocytes from circulation (Lang et al., 2005). In G6PD deficiency, this controlled phagocytic clearance mechanism may allow defective erythrocytes to escape intravascular hemolysis under mild stress conditions (Lang et al., 2012).

## Molecular crosstalk between ferroptosis and eryptosis in G6PD deficiency

Mature erythrocytes lack mitochondria and endoplasmic reticulum; they remain highly susceptible to lipid peroxidation due to the high polyunsaturated fatty acid (PUFA) content of their plasma membrane and the abundance of iron released from hemoglobin breakdown (Fortuna et al., 2024). In G6PD-deficient erythrocytes, eryptosis, ferroptosis, and erythronecroptosis are proposed to intersect, yet it is critical to recognize that the empirical evidence supporting these three regulated cell-death pathways is markedly unequal (Tkachenko et al., 2025).

Direct clinical and experimental evidence in G6PD-deficient human erythrocytes exists primarily for eryptosis, as demonstrated by accelerated phosphatidylserine exposure, cell shrinkage, and enhanced macrophage-mediated clearance (Bouguerra G, 2021). GPX4-dependent lipid peroxidation and ferroptotic signatures in stored red blood cells provide supporting relevance for iron-dependent membrane damage in non-nucleated erythroid systems (Stephenson et al., 2025).

The loss of NADPH production limits GR-mediated reduction of GSSG back to GSH (Turan et al., 2001). Because GPX4 requires GSH, limited NADPH indirectly impairs lipid peroxide clearance by slowing GSH regeneration. This mechanism explains higher lipid peroxidation, but it does not prove that ferroptosis occurs in mature erythrocytes (Yeerbao et al., 2026).

G6PD deficiency does not necessarily activate three independent death pathways. It may create a common redox environment in which different cell-fate programs become accessible.

## Erythronecroptosis

Erythronecroptosis is a newly recognized form of regulated necrosis in mature erythrocytes, driven by the classical receptor-interacting serine/threonine-protein kinase 1 (RIPK1), receptor-interacting serine/threonine-protein kinase 3 (RIPK3), and mixed lineage kinase domain-like protein (MLKL) signaling cascade rather than physical or osmotic trauma (Tkachenko and Havranek, 2025). Upon activation, phosphorylated MLKL forms oligomeric pores in the erythrocyte plasma membrane, leading to cell swelling, membrane rupture, and the systemic release of pro-inflammatory damage-associated molecular patterns (DAMPs) (Zheng et al., 2026). Consequently, this pathway converts erythrocyte death from a physiologically silent event into a potent driver of intravascular inflammation and vascular pathology (Cui et al., 2026).

There is currently no direct experimental data showing that G6PD deficiency or NADPH depletion activates the RIPK1/RIPK3/MLKL pathway in mature red blood cells. Because of this, erythronecroptosis should be treated strictly as a hypothesis that still needs experimental proof, rather than a proven feature of G6PD deficiency.

These distinct cell-death signatures, eryptosis, erythrocyte clearance, and hemolysis are not equivalent outcomes. Eryptosis promotes phosphatidylserine-mediated clearance of intact erythrocytes by macrophages, which may prevent intravascular rupture. Conversely, erythronecroptosis is intrinsically lytic, whereas the link between ferroptosis-like lipid peroxidation and membrane rupture in mature red blood cells remains incompletely defined. Rather than implying that all pathways inevitably cause hemolysis, it is more accurate to state that they contribute through distinct mechanisms to premature erythrocyte loss.

## Beyond hemolysis: redefining G6PD deficiency in erythrocyte redox control

For decades, G6PD deficiency has been viewed primarily as a hemolytic disorder (Author Anonymous, 1989; Luzzatto et al., 2020). However, hemolysis is the result rather than the underlying disease mechanism. Insufficient NADPH production disrupts cellular redox homeostasis, weakens antioxidant defenses, and promotes multiple regulated pathways of cell death, including eryptosis, ferroptosis, and erythronecroptosis. From this perspective, G6PD deficiency is better understood as a disorder of redox failure in which hemolysis is one terminal outcome. This shift turns the focus to upstream metabolic dysfunction to preserve redox balance and prevent erythrocyte loss.

## The redox checkpoint hypothesis

I present a testable conceptual model in which G6PD may act as a critical redox checkpoint controlling erythrocyte survival under oxidative stress. In healthy erythrocytes, G6PD continuously generates NADPH through the pentose phosphate pathway, thereby maintaining GSH, preserving GPX4 activity, and protecting membrane lipids from oxidative damage. This metabolic axis, G6PD-NADPH-GSH-GPX4, acts as a fundamental checkpoint that determines whether redox homeostasis can be maintained.

When G6PD activity declines because of inherited mutations or acquired metabolic inhibition, NADPH production may become insufficient to sustain antioxidant defenses.

When G6PD activity drops, NADPH production may fail to sustain cellular antioxidant defenses. The intracellular pool of reduced electron donors, specifically GSH and NADPH, plays a key role in regulating erythrocyte fate by neutralizing reactive species via enzymatic mechanisms (e.g., GPx activity) as well as direct non-enzymatic antioxidant scavenging (Daraghmeh and Karaman, 2024). Tandem mass spectrometry analysis in G6PD-deficient newborns has demonstrated a significant drop in GSH levels from 370 ± 53.2 μmol/L in healthy controls to 242.9 ± 15.9 μmol/L, with a GSH/GSSG ratio of 14.9 ± 7.2 (Gong et al., 2017). In healthy erythrocytes under physiological conditions, the GSH/GSSG ratio should be around 500:1 (Arese et al., 2012). Studies have established that adult human plasma levels typically range from 2 to 5.17 µM for GSH and around 0.14 µM for GSSG (Jones et al., 2000; Wang et al., 2012). The cells must maintain their NADPH/NADP^+^ ratio high enough for signaling pathways, biosynthesis, and antioxidant defense (approximately 100:1 in normal red blood cells) (Xiao et al., 2018). NADPH is required for the regeneration of GSH from its oxidized form, GSSG, via GR (Aydemir and Ulusu, 2024). Under severe oxidative load or enzymatic dysfunction or deficiency, NADPH depletion causes a severe decline in the GSH/GSSG ratio (Koju et al., 2022). Consequently, when lipid peroxides accumulate, catalase, peroxiredoxin-2 (Prx2), and GPx enzymes work in a coupled enzyme system linked to GSH and NADPH to detoxify endogenous and/or exogenous oxidants (Melo et al., 2024).

At present, no validated threshold for NADPH/NADP^+^ or GSH/GSSG ratios has been established to predict the transition from erythrocyte redox imbalance to eryptosis or hemolysis in G6PD deficiency. Reported values vary according to the biological sample, measurement method, and experimental or clinical conditions, limiting direct comparisons between studies. Therefore, these redox indicators should be interpreted as measures of antioxidant status rather than as established predictors of a specific erythrocyte fate. Further studies using standardized assays and well-characterized erythrocyte populations are needed to determine whether reproducible threshold values can be identified.

In this model, a critical reduction in redox buffering capacity could represent a threshold beyond which different erythrocyte fate responses become increasingly likely. However, the pathways should not be assumed to be coordinated or causally equivalent. Eryptosis has the strongest direct evidence in G6PD-deficient erythrocytes, whereas ferroptosis and particularly erythronecroptosis remain hypotheses requiring pathway-specific validation. The proposed G6PD-NADPH-GSH-GPX4 axis therefore serves as a testable metabolic framework rather than an established unifying mechanism. The proposed G6PD-NADPH-GSH-GPX4 axis serves as a testable metabolic framework illustrating how NADPH scarcity branches into distinct cell-death programs (Figure 1).

**FIGURE 1:**
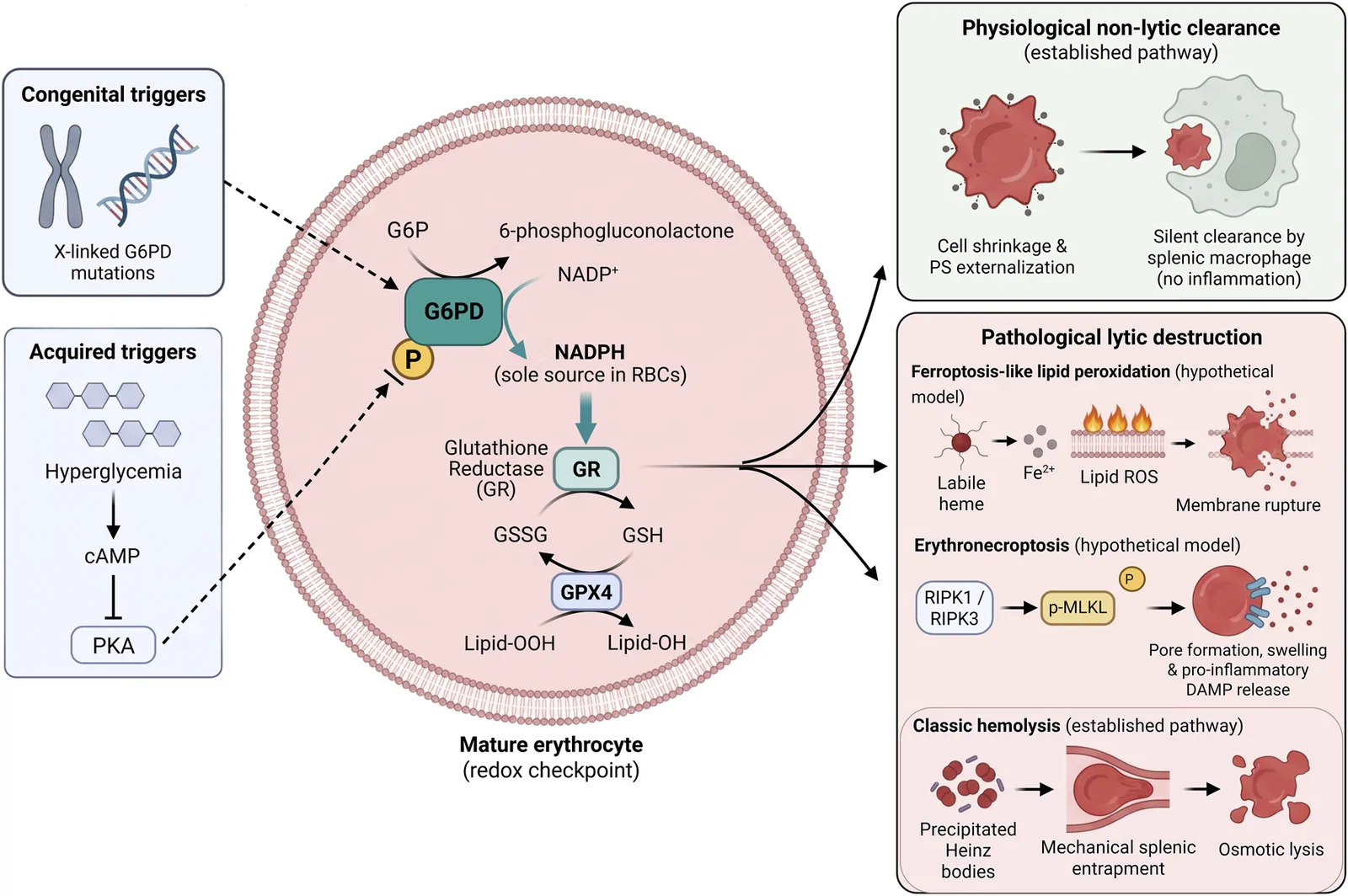
Overview of erythrocyte cell death pathways in G6PD deficiency. G6PD deficiency lowers NADPH levels, limiting GR and GPX4 activity. Without sufficient antioxidant support, erythrocytes undergo either non-lytic clearance (eryptosis) or lytic cell destruction, including ferroptosis-like damage, erythronecroptosis, and classic hemolysis. Created with https://BioRender.com.

The distinct biochemical features, membrane alterations, and antioxidant dependencies of these cell death pathways are summarized in Table 1.

**TABLE 1:** Comparative analysis of erythrocyte cell death pathways.

Feature	Eryptosis	Hemolysis (classic/Lytic)	Ferroptosis	Erythronecroptosis
Main mechanism	Programmed suicidal signaling (calcium influx, p38 MAPK)	Membrane rupture due to physical/osmotic stress or toxins	Iron ( ${\text{Fe}}^{2+}$ )-dependent lipid peroxidation	Programmed necrosis (RIPK1/RIPK3/MLKL pathway)
Key molecule	Phosphatidylserine (PS) externalization	Osmotic pressure/Toxins	Free iron and oxidized lipids (lipid ROS)	Phospho-MLKL, DAMPs release
Cell membrane	Shrinks, stays intact (engulfed by macrophages)	Ruptures (contents spill out into plasma)	Lipid bilayer collapses and ruptures	Swells, degrades, and ruptures (release of inflammatory DAMPs)
Antioxidant defense	Partially effective (accelerated by energy/ATP depletion)	Ineffective (sudden damage)	Regulated by GPX4	Ineffective once MLKL pore formation begins

## Sequential mechanisms and molecular components of the proposed model

Erythrocyte fate downstream of G6PD deficiency follows a defined sequence of biochemical events rather than a single catastrophic rupture. The core components of this checkpoint include the G6PD-NADPH axis, GR, GSH, and GPX4.

Although over 400 G6PD variants exist, the World Health Organization (WHO) classifies them based on residual enzymatic activity and clinical presentation. This classification reflects why the onset and severity of clinical severity, explaining why the onset and severity of clinical hemolysis vary significantly across different variants (Ulusu, 2024). In pathogenic variants or acquired deficiency, the initial molecular event is failure of NADPH regeneration (Beutler, 1994; Naville et al., 2022). This scarcity sequentially impairs GR activity, depletes cytosolic GSH pools, and secondarily disables GPX4-mediated clearance of lipid hydroperoxides.

Under oxidative stress, accumulating lipid hydroperoxides induce plasma membrane lipid peroxidation, a hallmark of ferroptotic injury (Feng et al., 2023). Concurrently, energy depletion and redox imbalance alter membrane asymmetry, triggering phosphatidylserine externalization (Nagata et al., 2016). This sequential cascade determines whether the erythrocyte enters physiologically silent macrophage-mediated clearance (eryptosis) (Tkachenko et al., 2025) or progresses to lytic membrane disruption. Under severe oxidative load, oxidized hemoglobin precipitates into Heinz bodies that impair membrane deformability. As splenic macrophages remove these rigid precipitates, the resulting bite cells and spherocytes undergo extravascular hemolysis, reinforcing the role of G6PD as a critical metabolic redox checkpoint (Zubair and Ravi B, 2026). The resulting bite cells and spherocytes undergo extravascular hemolysis, thereby strengthening the scientific validity of G6PD as a metabolic redox checkpoint.

## Conclusion

In conclusion, G6PD-dependent NADPH metabolism determines whether erythrocytes maintain redox balance or enter cell-death pathways. Viewing G6PD deficiency as a metabolic checkpoint rather than a simple cause of membrane lysis clarifies why erythrocytes undergo macrophage clearance under mild oxidative stress yet break down in circulation under severe conditions. While eryptosis is well supported by experimental evidence, the involvement of lipid peroxidation and erythronecroptosis in mature red blood cells requires further direct validation. Targeting upstream NADPH preservation rather than secondary membrane damage offers a rational strategy for protecting red blood cells in both inherited and acquired conditions.

## Future perspectives

Future treatments need to separate direct G6PD activation from broader cell signaling. Early upstream regulators, such as growth factors (PDGF, EGF, VEGF) and signaling proteins (PI3K, mTOR, TIGAR), modulate G6PD activity; however, targeting these pathways carries systemic risks, including oncogenic potential in nucleated cells (Stanton, 2012). Future research should prioritize the discovery of direct, selective small-molecule G6PD activators that safely restore enzymatic function in both inherited and acquired deficiencies without promoting off-target tumorigenesis.

In parallel, establishing standardized measurements of NADPH/NADP^+^ and GSH/GSSG ratios, alongside profiling key antioxidant enzyme activities and erythrocyte fate markers, will be essential to determine whether reproducible redox thresholds can predict specific cellular outcomes.

## Data Availability

The original contributions presented in the study are included in the article/supplementary material, further inquiries can be directed to the corresponding author.
